# Sudden Congestion and Death

**Published:** 1826-07

**Authors:** 


21. *Sudden Congestion and Death.* A man (Martineau) 42 years of age, of robust constitution and sanguineous temperament, was received into the Maison de Santé, of Charenton, on the 14th January, after having had several accessions of erotomania. These accessions came on once a month, and lasted a fortnight. In these attacks he became greatly agitated—talked incessantly—slept scarcely any—and ate very little. In the intervals he was tranquil; but his intellectual faculties were little developed. In this state he had continued a great many years. His physical health appeared perfectly good. On the 23d of June, two hours after breakfast, he walked out in the garden, the weather being beautiful, and the sun not too powerful. All at once he fell to the ground, insensible and motionless. He was raised up, and soon revived so far as to be able to walk for some minutes, when he again fell down, and never breathed or moved afterwards.

*Dissection.* The vessels of the brain and its membranes were more injected than natural; but there was no other appearance of disease throughout the encephalon. "The two lungs were gorged throughout with black and fluid blood." There was no other morbid appearance in any part of the body.

*Remarks.* The pulmonary congestion was not announced by any premonitory symptom. But Martineau had this time passed the usual period of his monthly attack of mania, and the author (M. Leuret) thinks that the congestion which periodically fell on the brain, producing the maniacal paroxysm, took the direction of the lungs this time, and caused instant death.—*Journ. General, Janvier*.

We believe that it is better to be in ignorance than in error. In the former case, our mind is open to truth when it presents itself—in the latter, it is too often secure in its false position, and seeks not to move from thence. We are doubtful whether death, in the above instance, was owing to the pulmonary congestion found after death. The patient fell down apparently dead a few minutes before, and soon recovered so as to walk about again. Could the pulmonary congestion have *then* obtained, and so suddenly vanished? The thing is next to impossible. And if so, why might not the same cause, whatever it was, which produced the first loss of sense and motion, produce also the second attack, which ended in death? We are more inclined to look to the sensorium for this sudden extinction of the vital functions than to the lungs True it is, there was only a slight injection of vessels found in the brain; but this is no proof that great lesion of the sensorial *functions* had not previously taken place. We see that, for a great many years, there was a regular monthly attack of mania, lasting for thirteen or fourteen days each paroxysm, and, after all, leaving no trace of organic disease or alteration of structure in any part of the encephalon, or its membranes. Why then should not this disturbance of function rise to a height incompatible with life, and leave nothing more than a little fullness of vessels—nay, not even so much as that? The case is instructive in respect to the pathology of insanity—proving that aberration of intellect may take place for years, without appreciable alteration of structure in the seat of thought. Hence we have every reason to conclude, that those organic changes, which we find in the heads of maniacs, are more frequently the effects than the causes of deranged sensorial functions. This reasoning, indeed, will apply to most of the diseases of the body as well as those of the mind.

